# Pore Structure Characteristics of Vegetated Concrete and Their Influence on Physical Properties

**DOI:** 10.3390/ma19051042

**Published:** 2026-03-09

**Authors:** Fazhi Huo, Xinjun Yan, Jiaqi Liu, Peiyuan Zhuang

**Affiliations:** 1 College of Water Conservancy and Civil Engineering, Xinjiang Agricultural University, Urumqi 830052, China; hfz_xjau@163.com (F.H.);; 2 Xinjiang Key Laboratory of Hydraulic Engineering Security and Water Disasters Prevention, Urumqi 830052, China

**Keywords:** vegetated concrete, porosity, fractal dimension, compressive strength, permeability coefficient

## Abstract

In this study, CT scanning technology was combined with ImageJ 1.54r and Avizo 3D 2022 professional image analysis software to quantify porosity. The aim was to reveal the intrinsic correlation between the pore structure characteristics and the macroscopic properties of vegetated concrete. A combination of 3D reconstruction, fractal analysis and multi-parameter regression modelling techniques was utilised to quantify the association between pore parameters and material properties. The mechanistic role of pore structure in regulating the strength–permeability trade-off relationship was elucidated. The results show that: (1) aggregate particle size and porosity are significantly negatively correlated with the compressive strength of vegetated concrete and strongly positively correlated with the water permeability coefficient, while the effects of both of them on the pH value of the material are negligible; (2) the porosity obtained by the image analysis method meets the design requirements of the target porosity, and the deviation between the computed 3D porosity from CT scanning and the 2D sliced porosity is less than 1%. The image analysis porosity is slightly lower than the measured value, a deviation within a reasonable range. (3) There is a robust positive correlation between the fractal dimension of the vegetated concrete structural surface and porosity. With increasing aggregate size, porosity gradually increases, pore network connectivity is significantly enhanced, and the fractal dimension increases correspondingly. (4) Function fitting analysis confirms that the correlation between the connected porosity and the compressive strength and permeability coefficient is more significant than that of the cross-sectional porosity. Specifically, compressive strength is significantly negatively correlated with equivalent pore size and fractal dimension, and the water permeability coefficient is strongly positively correlated with these two parameters. This study can provide important theoretical support and engineering reference for the optimization of the mix proportion and performance control of vegetated concrete.

## 1. Introduction

Ecological civilisation construction is a national strategy and social practice that promotes the harmonious coexistence of humans and nature, with sustainable and green development at its core [1]. In river training and other water conservancy projects, traditional concrete berms are usually poured with full-section closure. This destroys the material cycle and energy exchange channels between water, organisms and soil, significantly weakening the self-purification ability and natural recovery function of river ecosystems [2]. Vegetated concrete, a green material providing both engineering protection and ecological services, is mainly composed of a porous concrete matrix, a surface vegetation substrate, and a vegetation system [3]. Its main advantage is that it provides sufficient water, nutrients and root expansion space for plant growth through an internal connected pore network, thus overcoming the ecological barrier of traditional concrete [4,5]. When used in slope protection projects, porous concrete is employed as the primary protective material in the initial stage. As the surface vegetation grows and develops, its root system can penetrate the concrete pores and extend to the bottom soil, gradually forming an ecological protection system in which vegetation communities are the main component. Over time, weathered porous concrete transforms into a surface cover layer, further enhancing the slope’s resistance to wind and rain erosion. Ultimately, this process realises the ecological transformation of the hardened project surface and establishes vegetation concrete as the core technology in the construction of water conservancy ecological projects.

The macroscopic physical properties (e.g., compressive strength and water permeability) and long-term durability of vegetated concrete depend primarily on the characteristics of its internal pore structure. Key evaluation parameters include porosity, equivalent pore size, distribution and connectivity [6,7,8,9]. Understanding the distribution characteristics of the pore structure is essential for matching the macroscopic and microscopic performance of vegetated concrete, optimising plant selection and adaptive design, and revealing the growth and development patterns of roots within the pore space [10]. However, the internal pore structure of vegetated concrete is highly complex, random and spatially heterogeneous. Traditional detection methods struggle to accurately quantify and systematically analyse pore parameters, which restricts the optimal design of its performance and the promotion of its large-scale engineering application.

The continuous porosity of vegetated concrete typically ranges from 20% to 30% [6,11]. Chen J. et al. [12] investigated the relationship between aggregate particle size and the properties of vegetated concrete and demonstrated that the enhancement of aggregate particle size was accompanied by an increase in the porosity and pore size of the concrete. Sumanasooriya et al. [13] examined the effect of different admixtures on the pore structure of vegetated concrete, permeability and strength and found that the addition of admixtures positively affected the permeability and pore structure, although it reduced the compressive strength of the concrete. Torres A [14] suggested that the performance of vegetated concrete is closely linked to its pore structure characteristics, which include coarse aggregate particle size, pore volume, pore size, pore size distribution, and cement dosage.

The cross-fertilization of digital image processing technology and fractal theory provides an effective technical means for the fine characterization of the internal structure of porous materials. Scholars globally have conducted numerous studies on the correlation between the pore characteristics and performance of porous concrete, achieving a series of results. MS Sumanasooriya et al. [15] proposed a methodology for determining the pore structure of vegetated concrete through the utilisation of two-dimensional image processing techniques. Chung et al. [16] successfully reconstructed new vegetated concrete specimens exhibiting diverse pore distributions through the employment of CT scan images and low-order probability functions. It was ascertained that the pore distributions of the reconstructed specimens were statistically analogous to the original specimens and possessed analogous physical properties. Numerical simulation tests indicated that the actual test cycle could be significantly shortened by reconstructing the specimens. Chen Z. et al. [17] investigated the pore characteristics of permeable concrete based on 2D and 3D CT image recognition and analysed the link between pore characteristics and permeability. Kuang et al. [18] extracted the porosity of permeable concrete by X-ray computed tomography (CT) scanning images, continuous porosity parameters such as pore tortuosity and pore diameter distribution, and proposed an optimised Kozeny-Carman model to verify the relationship between permeability coefficient and porosity. Zhang et al. [19] constructed a three-dimensional structural model of permeable concrete by using the three-dimensional reconstruction technique of CT images, analysed the connecting pore network and the target pore space, and implemented the seepage simulation. Wei et al. [20] constructed a fractal model of the spatial structure of concrete paste based on fractal theory and derived a quantitative correlation equation between the fractal dimensions, porosity, and distribution of pore characteristics, providing theoretical support for the quantitative characterization of pore structure. Liang et al. [21,22], to meet the demand for accuracy in three-dimensional detailed mechanical simulation of porous concrete, used CT and three-dimensional reconstruction principles, combined with digital image processing technology and MATLAB programming, to accurately reconstruct the internal detailed structure, laying a technical foundation for studying the evolution mechanism of material properties at the mesoscale. Jiang et al. [23] used digital image processing technology to directly observe the internal pore size distribution and surface porosity characteristics of porous concrete, quantitatively revealing the regulatory mechanisms of key factors such as pore size, water–cement ratio, and aggregate particle size on pore size distribution patterns and surface porosity connectivity. Zeng et al. [24] sectioned vegetated concrete and systematically reconstructed its internal fine structure using image processing platforms such as PS, MATLAB, and ImageJ. They systematically analyzed the morphology of cross-sectional pores, compared image-analysis porosity with measured porosity, calculated the fractal dimension of the pore network using the box-counting method, and established a functional correlation model of pore structure characteristics and fractal dimensions. Yin et al. [25] used CT scanning technology to capture the three-dimensional damage evolution characteristics of the internal pore structure of vegetated concrete under freeze–thaw cycles, elucidating the intrinsic mechanism of material performance deterioration in a freeze–thaw environment. Zhang et al. [26] found, through a comparative test of eight different gradations (single-sized, gap-graded, and continuously graded) of permeable concrete, that continuously graded specimens exhibited low porosity, a small permeability coefficient, and high compressive strength. Kusumawardani et al. [27] used shape factor and sphericity index to quantify aggregate shape characteristics and confirmed that aggregate shape significantly affects the overall performance of porous mixtures, with performance showing a decreasing trend as aggregate sphericity increases.

The preceding studies establish the correlation between pore structure and the macroscopic properties of porous concrete. However, the majority of extant studies have concentrated on total porosity as the key pore structure parameter, with less attention paid to connected porosity and its differential impact on material properties. Secondly, although fractal theory has been utilised to characterise pore complexity, the direct correlation between fractal dimension and strength and permeability remains to be fully elucidated. The quantitative mechanisms by which aggregate size regulates the evolution of pore structure (including pore size distribution, connectivity, and fractal characteristics), and thus influences macroscopic properties, remain underexplored. This study therefore combines macroscopic physical property testing with CT scanning and 3D reconstruction technology to systematically analyse the characteristics of the fine pore structure of vegetated concrete. The aim is to construct quantitative fitting relationships between pore characteristic parameters, fractal dimensions and macroscopic properties, and to clarify the regulatory mechanism of aggregate particle size on the key properties of vegetated concrete. These results could provide the theoretical basis and technical guidance for optimising the proportions of vegetated concrete mixes, precisely controlling their performance, and efficiently applying them to water conservancy ecological slope protection projects.

## 2. Materials and Methods

### 2.1. Test Materials

Cement: Ordinary Portland cement of grade 42.5 was used in this test, and its physical and mechanical properties are shown in Table 1.

**Table 1:** Main Physical Properties of Cement.

Density (kg·m^−3^)	Standard Consistency (%)	Specific Surface Area (m^2^·kg^−1^)	Flexural Strength (MPa)		Compressive Strength (MPa)		Setting Time (min)	
			3 d	28 d	3 d	28 d	Initial Condensation	Final Condensation
3040	27	384	5.4	6.5	27.3	42.5	172	230

Fly ash: The fly ash used was Classifly ash from the Guizhou Nayong power plant. Its properties are shown in Table 2.

**Table 2:** Physical Properties and Chemical Composition of Fly Ash.

Fineness (%)	Moisture Content (%)	Loss on Ignition (%)	SiO_2_ (%)	Al_2_O_3_ (%)	CaO (%)	SO_3_ (%)
8.2	0.1	4.1	51.47	36.27	6.7	0.38

Coarse aggregate: In this study, single-sized (uniform) crushed stone aggregates with particle size ranges of 10–15 mm, 15–20 mm, and 20–25 mm were utilised to achieve the high interconnected porosity that is required for vegetated concrete applications. Their properties are shown in Table 3.

**Table 3:** Crushed Stone Performance Indicators.

Pebble Size (mm)	Packing Density (kg·m^−3^)	Compact Packing Density (kg·m^−3^)	Apparent Density (kg·m^−3^)	Bulk Density in the Loose State (%)	Bulk Density in the Compacted State (%)
10~15	1522	1599	2625	42.02	39.09
15~20	1477	1560	2635	43.95	40.80
20~25	1438	1554	2630	45.32	40.91

Admixture: Admixture adopts Nanjing manatee permeable concrete enhancer. The addition of this compound enhancer ensures that the vegetated concrete prepared by the slurry wrapping method achieves the dual control targets of mechanical properties and pore structure in this study. It ensures that the cement paste coats the coarse aggregate evenly and stably, avoids pore blockage caused by paste segregation and loss, and maintains the designed connected porosity and water permeability. It also significantly improves the interface bonding strength between the aggregate and the paste. Their properties are shown in Table 4.

### 2.2. Preparation Method and Mixing Ratio Design

The casting method, the shelling method and the slurry method are three of the most common methods used to prepare vegetated concrete. The slurry method can effectively improve the workability of the concrete, enhancing its strength and durability; this is why it was adopted in this experiment [3]. Before formal mixing, a premixing process was implemented in which cement, fly ash, crushed stone and a reinforcing agent were placed in the mixer for one minute to achieve initial homogenisation. Then, mixing water was added and the materials were wet-mixed for three minutes. This process can significantly reduce the loss of cementitious materials and mixing water, prevent adhesion to the inner wall of the mixing chamber and ensure accurate proportions. The final mix resulted in the cementitious materials fully coating the aggregates, giving a uniformly distributed, moist appearance with no defects such as exposed aggregates or cementitious agglomerates. The vegetated concrete specimens were filled in layers and vibrated. Immediately after vibration, they were covered with a film for three days to control water evaporation. After demoulding, the specimens were transferred to a standard curing environment (temperature of 20 ± 2 °C, relative humidity ≥ 95%) until performance testing at 28 days.

The project requires the compressive strength of the vegetated concrete to be no less than 5 MPa, with a continuous porosity of no less than 20%. If the design porosity exceeds 30%, the cementitious material in the vegetated concrete will sink. Therefore, formal testing determined a design porosity range of 20% to 30%, centred at 25%.

This test used target porosity as the control parameter to effectively regulate the porosity of the vegetated concrete. Therefore, the volumetric method was employed for the design of the mixture proportions. Pre-tests determined the optimal water–cement ratio to be 0.3, and the fly ash replacement rate for cement was found to be 26%. Nine groups of tests were designed by varying the aggregate particle size and target porosity. The test mixes are shown in Table 5.

### 2.3. Test Methods

For each test method (compressive strength, alkalinity, porosity, permeability coefficient, and CT scan), three replicate specimens were prepared and tested for each fit ratio.

#### 2.3.1. Compressive Strength

The compressive strength test was conducted in accordance with the relevant provisions of the ‘Standard for Test Methods of Physical and Mechanical Properties of Concrete’ (GB/T 50081-2019) [28], with the loading rate controlled between 0.3 and 0.5 MPa/s. One side of each specimen was selected as the compression surface, ensuring it was flat and free of protrusions. The test was performed using a STYE-2000J rock shear and compression testing machine (Zhejiang Geotechnical Instrument Manufacturing Co., Shaoxing, China).

#### 2.3.2. Alkalinity Test

The solid–liquid extraction method [29] was employed. Concrete test blocks that had reached the required age were crushed. The cementitious material was collected and ground sufficiently before being passed through a 0.074 mm standard test sieve. Five grams of the powder were weighed and mixed with 50 g of distilled water in a test tube. The tube was tightly sealed with a rubber stopper to prevent carbonation and shaken every five minutes. After 2 h, the mixture was filtered through filter paper. The pH value of the resulting aqueous solution was then measured using a pH-100A pen-type acidimeter (Shanghai Yueping Scientific Instrument Co., Ltd., Shanghai, China).

#### 2.3.3. Porosity Test

The pores in vegetated concrete consist of open and closed pores. It is the open pores that mainly determine its water permeability. Porosity is a key parameter of pore structure in porous materials and is not only the main index for measuring water permeability, but is also closely related to the material’s mechanical properties. Cross-sectional porosity is defined as the ratio of the total pore area to the cross-sectional area of the concrete. The calculation formula is as follows:

(1) $$\bar{p_{1}}=\frac{1}{n}\sum_{j=1}^{n}\left(\frac{\sum_{i=1}^{N}A_{ij}}{A_{1}}\right)\times 100\%$$

where: *P*_1_ is the cross-sectional porosity; *A_i_* is the area of the ith pore on the cross-sectional image; *A*_1_ is the total area of the cross-sectional image.

The effective porosity was measured by water displacement method. The sample was dried in a desiccator at 110 °C for 24 h and then placed in water for 24 h to obtain the volume of water displaced by the sample, *V_d_*. The effective pore volume was calculated by subtracting *V_d_* from the total sample volume, *V_b_*. The effective porosity of the sample was calculated as follows:

(2) $$P=\left(\frac{V_{b}-V_{d}}{V_{b}}\right)\times 100\%$$

#### 2.3.4. Permeability Coefficient

**Figure 1:**
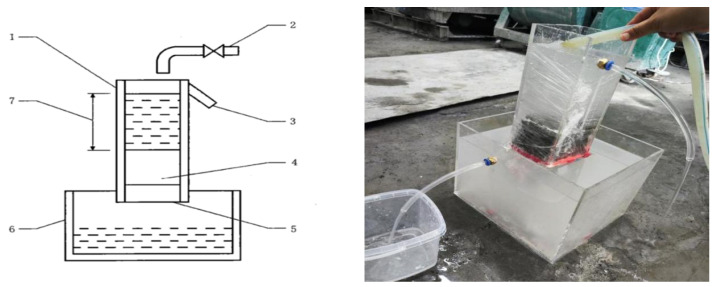
Permeability Coefficient Testing Apparatus. Note: 1—Column sealing device, 2—Water supply system, 3—Overflow outlet, 4—Test piece, 5—Water outlet, 6—Measuring container, 7—Water level difference.

The constant head method was chosen due to the large pore sizes characteristic of vegetated concrete. Compared with the falling head method, which can result in insufficient controllability of the test time, the constant head method is more suitable for determining the permeability coefficient of vegetated concrete due to its significant pore connectivity and the advantage of a stable hydraulic gradient. The test was carried out in accordance with Appendix A of JC/T 2558-2020, ‘Permeable Concrete’ [30]. The specific steps were as follows: the perimeter of the test block was completely sealed with cling film. The sealed test block was placed in the apparatus and the contact position was sealed with rubber cement to prevent water leakage during the test. The water supply valve was opened to fill the container with water, and the intake was adjusted to maintain a constant water level of approximately 150 mm in the prismatic sealing device. Once the flow from the overflow outlet had stabilised, the outflow was collected using a measuring instrument. The amount of water discharged over 90 s was recorded and the measurement repeated three times to obtain an average value. As shown in Figure 1. The permeability coefficient was then calculated using the following formula:

(3) $$K_{T}=\frac{Q\cdot L}{A\cdot H\cdot \;t}$$

where: *K_T_* is the water permeability coefficient at water temperature *T* °C, mm/s; *Q* is the amount of water flowing out at time t seconds, mm^3^; *L* is the thickness of the specimen, mm; *A* is the surface area of the specimen, mm^2^; *H* is the difference in water level, mm; *t* is the time of determination, s.

#### 2.3.5. Hole Structure Image Acquisition

**Figure 2:**
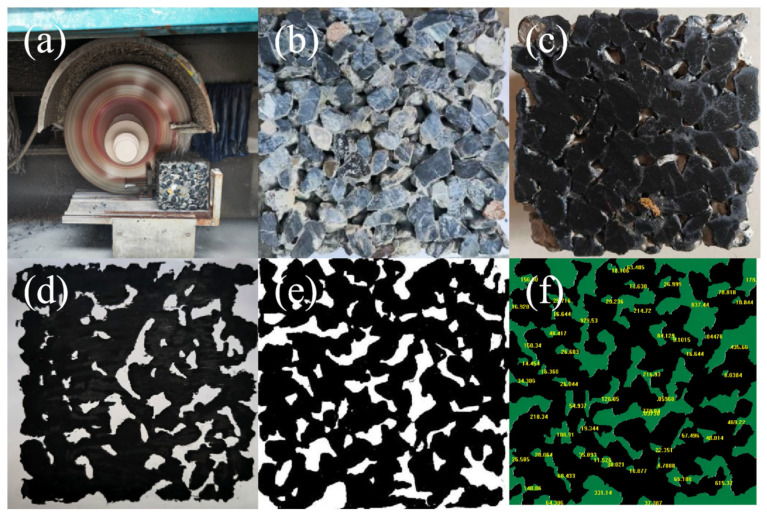
Acquisition of Vegetation Concrete Pore Morphology (**a**) Machine cutting; (**b**) Original section; (**c**) Pigment brushing; (**d**) Structure rubbing; (**e**) Threshold segmentation; (**f**) Pore distribution statistics.

A rock cutter was used to obtain vegetated concrete profiles at various depths. Pigment was then applied evenly to the cut surface using a brush. A layer of white A4 paper was pressed onto the inked surface to create a topographical print of the pore structure. This was then photographed with a camera and the image transferred to a computer for processing. For image processing, a threshold segmentation method was adopted: the image was segmented into two regions (pores and fillers) by setting a grayscale threshold. The grayscale image was then binarised and filtered in MATLAB R2024b to classify pixels of different grayscales into two categories: white for pores and black for fillers, such as paste and aggregate. Finally, Image-Pro Plus 6.0 software was used to further process the images to obtain planar porosity and pore size frequency distributions, as shown in Figure 2.

#### 2.3.6. Equivalent Pore Size Test

The equivalent pore diameter is used to analyse the size and distribution of pores inside the vegetated concrete, in order to demonstrate its pore gradation characteristics. An equivalent pore diameter of more than 0.8 mm was selected as a screening condition to eliminate minor identification errors during data pre-processing. The equivalent pore diameter is defined as the diameter of a circle with the same area as the target pore and is calculated as follows:

(4) $$D_{p}=2\sqrt{\frac{A}{\pi }}$$

where *D_p_* is the equivalent pore diameter, mm; *A* is the pore area, mm^2^.

#### 2.3.7. Fractal Dimension Test

The fractal dimension is a key measure of the self-similar nature of fractal structures and reflects the geometric complexity of irregular structures. In pore structure studies, this parameter is a key indicator used to characterise the heterogeneity of pore topology. A fractal dimension value closer to 2 indicates greater structural irregularity in the pore system.

This paper uses the easily implemented box-counting dimension [31,32] to depict the complexity of the pore structure. It is calculated as follows:

(5) $$D=\underset{r\to 0}{\lim}\frac{\mathrm{lg}N(r)}{\mathrm{lg}r}$$

where: *r* is the box size; *N*(*r*) is the number of non-empty boxes covering the graph.

#### 2.3.8. X-Ray CT Scanning and 3D Reconstruction

**Figure 3:**
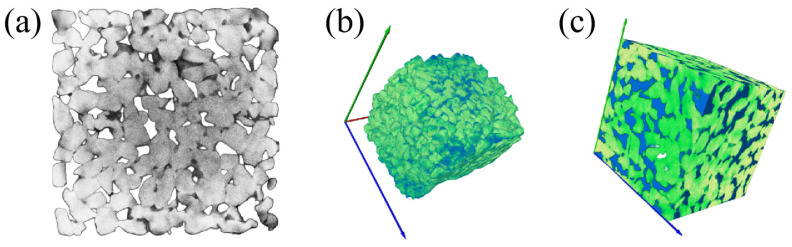
Microscopic Structure of Three-Dimensional Models (**a**) Original two-dimensional CT slice; (**b**) Three-dimensional re-constructed model; (**c**) Three-dimensional model after sectioning.

This study used a high-resolution industrial CT system (Phoenix V|Tome|X S, Waygate Technologies, Wunstorf, Germany) to scan vegetation-reinforced concrete specimens using X-ray computed tomography, enabling the non-destructive characterisation of their internal pore structures. Cubic specimens measuring 150 mm on each side were selected for CT scanning. As shown in Figure 3. The core region was designated as the scanning volume in order to eliminate defects in the surface layer caused by the forming process. The key scanning parameters were set as follows: X-ray tube voltage: 180 kV; tube current: 150 μA; integration time per projection: 300 ms. During a full 360° rotation, 1080 projection images were acquired at 0.33° increments. The reconstructed tomographic images featured isotropic voxel dimensions of 45 μm.

Image reconstruction employed the Feldkamp–Davis–Kress filtered backprojection algorithm. Preprocessing steps included annular artefact correction and Gaussian low-pass filtering to reduce image noise while preserving the edge features of the pore–agglomerate interface. The reconstructed 2D slice series was imported into Avizo for 3D visualisation and quantitative analysis.

Quantitative image segmentation used the Otsu adaptive thresholding method to distinguish three phases within the tomographic images: coarse aggregates, cement paste and pore space. The CT image grayscale histogram exhibits three distinct peaks corresponding to these phases. The optimal segmentation threshold between adjacent phases was automatically determined by maximising the inter-class grayscale variance. Following segmentation, the total pore network, connected pore network and closed pore network were reconstructed as 3D models using the voxel counting method. Pore structure parameters, including 3D porosity, pore connectivity and equivalent pore size, were calculated quantitatively.

## 3. Results and Analysis

### 3.1. Macro-Physical Properties

**Figure 4:**
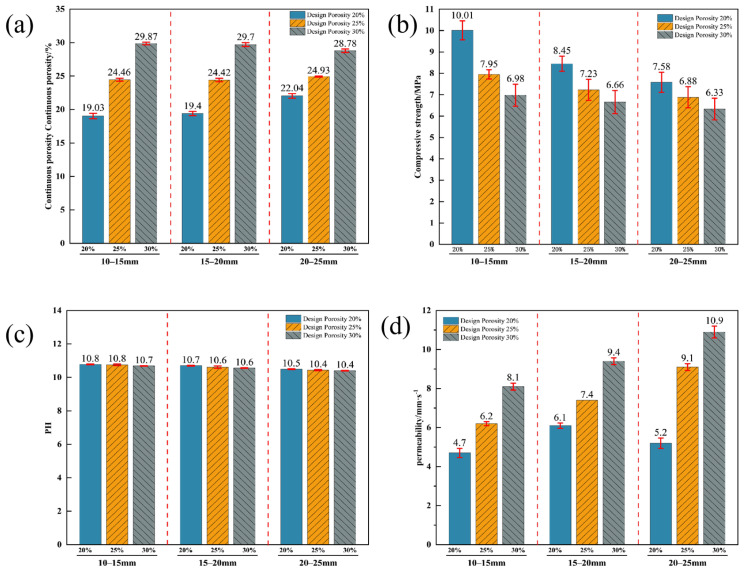
Macroscopic Properties of Vegetated Concrete with Different Particle Sizes and Porosities (**a**) Continuous porosity; (**b**) Compressive strength; (**c**) pH value; (**d**) Permeability coefficient.

The results of macro-physical properties of vegetated concrete are shown in Figure 4. A statistical analysis showed that the *p*-values were less than 0.01, indicating a significant effect of aggregate particle size and design porosity on its physical properties.

Changes in the size and porosity of aggregates have a significant impact on the physical and mechanical properties of vegetated concrete. As Figure 4 shows, the measured porosity, compressive strength and water permeability coefficient are significantly affected by aggregate particle size and target porosity, while their effect on pH is relatively small. For a given particle size, porosity is negatively correlated with compressive strength and positively correlated with the water permeability coefficient. At similar porosity levels, an increase in aggregate particle size leads to a decrease in compressive strength, while the water permeability coefficient increases significantly. Increasing both aggregate particle size and porosity leads to a slight decrease in pH value.

The measured effective porosity is slightly lower than the target porosity due to the presence of closed pores. For the 20–25 mm particle size group with a target porosity of 20%, the porosity setting was too low. This resulted in the cement paste not adhering completely to the aggregate surface, causing slight sedimentation. Consequently, when the effective porosity was determined using the water displacement method, the volume of displaced water decreased, leading to a higher measured porosity. Simultaneously, sedimentation blocked some connected pores, resulting in a decreased water permeability coefficient. As the target porosity increased, the deviation of the effective porosity from the target value for each particle size group decreased gradually.

The strength of vegetated concrete is primarily determined by the ability of the cement paste to coat and bond the aggregates. Improved strength largely depends on the cement paste’s bonding ability between aggregate particles, which in turn is affected by the paste’s cohesion and contact area with the particles. Cement paste that is stronger and thicker can effectively enhance the structural integrity of vegetated concrete.

### 3.2. Porosity

As can be seen from Table 6, the measured porosity varies among the vegetated concrete specimens and the connected porosity is consistently smaller than the cross-sectional porosity. This is partly because the porosity obtained from cross-sections includes closed pores that cannot be measured when connected porosity is determined experimentally. It may also be due to edge stones or cementitious materials being dislodged during cutting and grinding, which increases the measured cross-sectional porosity.

**Figure 5:**
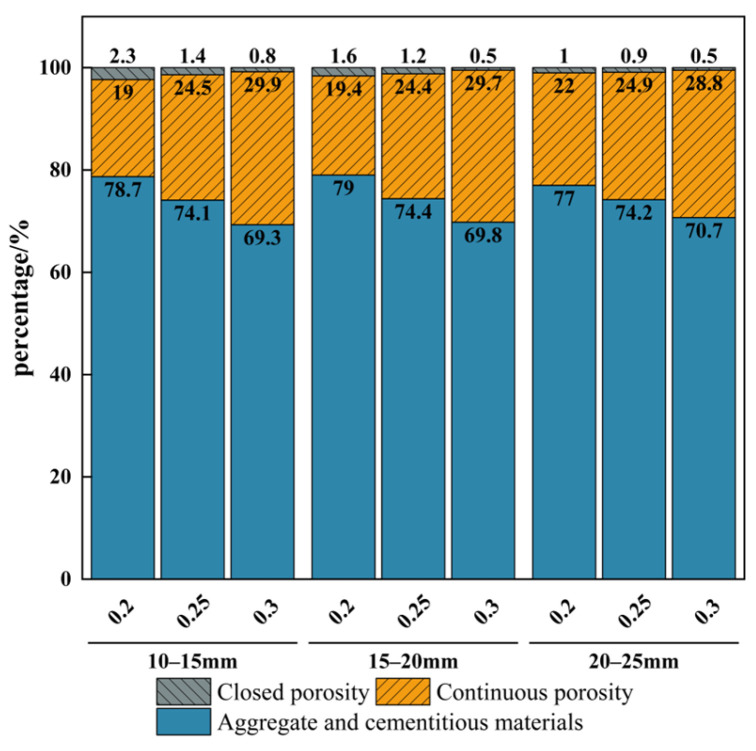
Percentage of each component for specimens with different particle sizes and porosities.

As Figure 5 shows, the planar porosity distribution of the vegetated concrete indicates that the size of the aggregate particles and the target porosity significantly impact the pore structure, especially the proportions of connected and closed pores. When the target porosity was high, the closed porosity was relatively low. Specimens with larger aggregate sizes generally exhibited lower closed porosity. This is mainly because an increase in aggregate particle size reduces the amount of cementitious material relative to the aggregate. The cementitious layer coating the aggregate surface becomes thinner, facilitating the formation of a connected pore structure. The physical support provided by the aggregates reduces the demand for cementitious materials and promotes pore interconnection. Therefore, larger aggregate sizes improve the permeability of vegetated concrete.

By contrast, the proportion of closed pores increased significantly when smaller aggregates were used, which limited the permeability of the vegetated concrete. This is because smaller aggregates are more densely packed within the matrix, forming a closed structure between pores and decreasing the number of connected pores. As the target porosity increased, the proportion of closed pores decreased, particularly in specimens with small-sized aggregates.

### 3.3. Equivalent Pore Size

**Figure 6:**
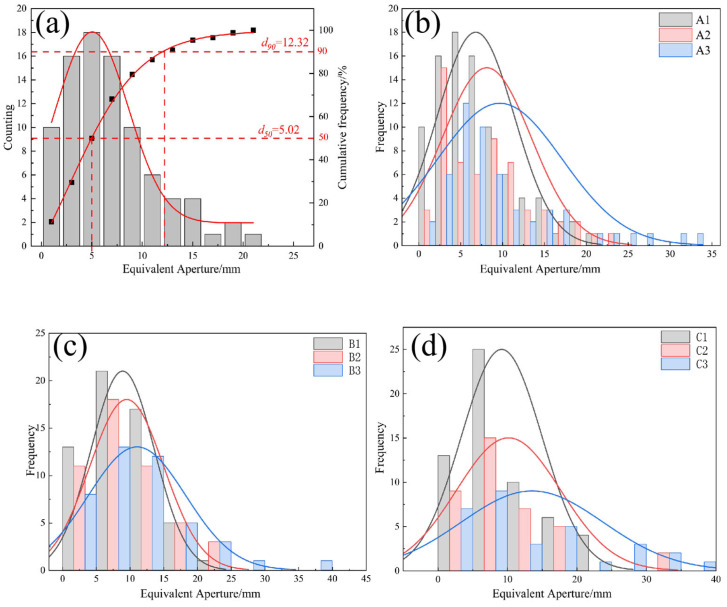
Histogram of the Probability Distribution of Equivalent Pore Diameters for Different Particle Sizes and Porosities (**a**) A1 Group; (**b**) A Group; (**c**) B Group; (**d**) C Group.

The pore size data can be imported into statistical analysis software to calculate the percentage of equivalent pore sizes within each specimen, as well as to plot their normal distribution curve. Figure 6 shows the equivalent pore sizes for specimens with different mix proportions, derived from these curves.

As Figure 6a shows, the pore diameters in group A1 followed an approximately normal distribution. However, they had a large kurtosis coefficient and a clear left skew, indicating a significant trend towards data concentration. Smaller pores (<10 mm) accounted for up to 79% of the total pore count, with dominant pores concentrated in the 5 mm interval forming a main peak in the distribution. The characteristic pore size values are d_50_ = 5.02 mm and d_90_ = 12.32 mm. This indicates that the material’s internal structure is dominated by a network of microscopic pores, which has a decisive influence on its permeability characteristics.

As can be seen from the figures above, the equivalent pore diameters for specimens with aggregate sizes of 10–15 mm, 15–20 mm and 20–25 mm are concentrated within the ranges 2–8 mm, 5–12 mm and 8–15 mm, respectively. As the porosity of the vegetated concrete increases, the centre of gravity of the histogram shifts gradually to the right and the normal distribution curve changes gradually from ‘tall and thin’ to ‘short and stout’. This indicates that, as porosity increases, so does pore size, with the proportion of large pores rising accordingly and the pore distribution becoming more uniform. Furthermore, as aggregate size increases, the proportion of large pores in the sample rises and pore distribution across different size ranges becomes more uniform. This is because larger aggregates will form larger pores during natural packing under otherwise identical mix proportion conditions, making it easier to create larger pores.

### 3.4. Fractal Dimension

In the study of vegetated concrete, measured porosity is a key indicator to characterize the macroscopic features of the specimen, while fractal dimension is used to quantify the complexity, irregularity and self-similarity of its pore structure. Establishing the quantitative relationship between aggregate particle size, porosity and fractal dimension is the basis for in-depth understanding of the performance indexes of vegetated concrete.

**Figure 7:**
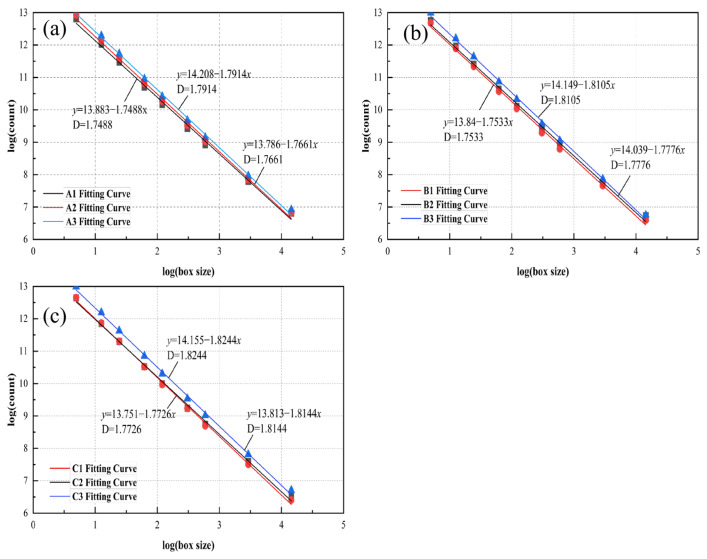
Pore Meter Box Dimensions (**a**) A Group; (**b**) B Group; (**c**) C Group.

The fractal dimension of the pores was calculated by Image J 1.54r image analysis software, and the obtained data were imported into Origin for fitting, so as to obtain the fractal dimension, and the results are shown in Table 5. It can be seen from Figure 7 that the lg N(L)–lg(1/L) curves of the vegetated concrete strictly follow the linear relationship, which indicates that the pore structure of the concrete has a fractal characteristic in a certain scale range, and the straight line of the slope is the fractal dimension of the concrete pore characteristics. The test results show that the fractal dimension tends to increase with the larger measured porosity and aggregate size of the vegetated concrete specimens. According to the fractal theory, the larger the fractal dimension is, the more complex the pore size and spatial distribution of pores in the vegetated concrete is, and the stronger the ability of pores to occupy space. Therefore, the pore structure of vegetated concrete possesses fractal characteristics. By establishing the relationship between aggregate particle size/measured porosity and pore fractal dimension, an important link between the macro and microscopic properties of this material and the fractal theory was effectively constructed.

### 3.5. Detailed Structure of the 3D Model

**Figure 8:**
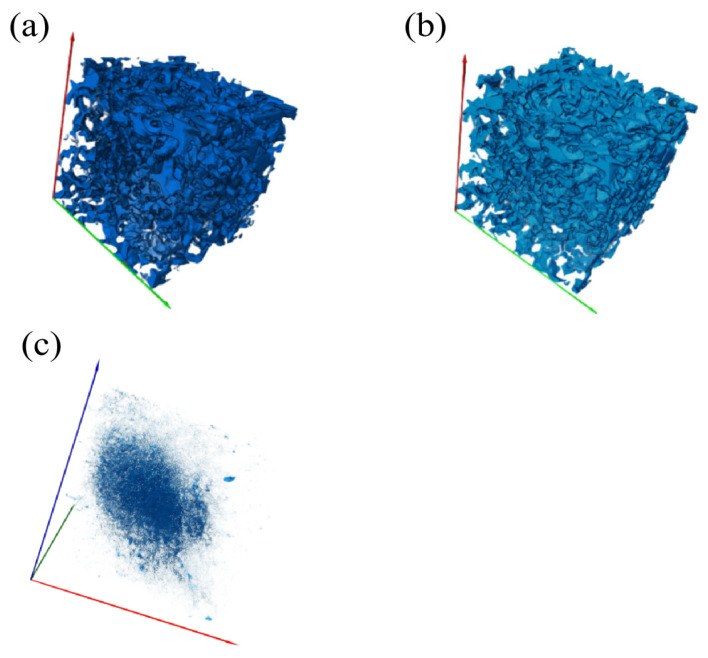
Three-dimensional pore distribution map (**a**) Total Porosity 3D Map; (**b**) 3D diagram of interconnected pores; (**c**) Closed-Pore 3D Diagram.

A three-dimensional pore space distribution model of the vegetated concrete was extracted based on CT image processing and analysis technology, including the total pore network, the connected pore network and the closed pore network (taking Group A as an example), as shown in Figure 8. The total porosity of Group A specimens was calculated using the voxel counting method to be 21.02%, 26.57% and 31.74% respectively. This is essentially consistent with the porosity obtained from 2D planar image analysis. This demonstrates the reliability of the voxel counting method for determining 3D porosity.

Using a three-dimensional spatial topology analysis combined with a 3D connectivity discrimination algorithm, the connected porosities of Group A specimens were measured to be 17.62%, 23.16% and 28.67% respectively. These values differ from the experimentally measured connected porosities of 18.03%, 23.46%, and 29.17% by 0.41%, 0.30%, and 0.50%, respectively. The main sources of error are twofold: first, non-saturated water penetration through microcracks and surface defects during testing may lead to higher measured values, and second, the pore connectivity criterion used in CT image processing may filter out some effective connected channels.

**Figure 9:**
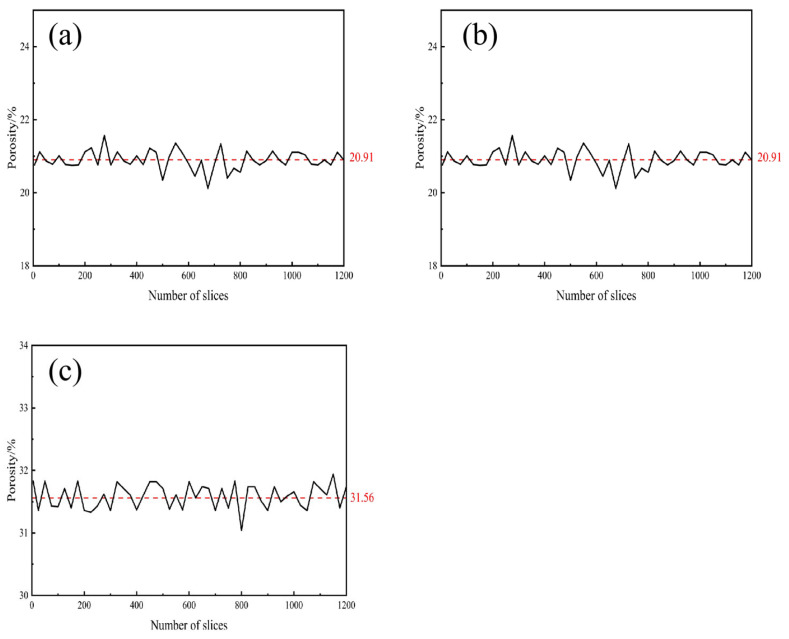
Porosity Distribution Diagram for Different Slice Layers in Group A (**a**) A1 Group; (**b**) A2 Group; (**c**) A3 Group.

The planar porosity distribution characteristics of Group A specimens along the depth direction are illustrated in Figure 9. In order to eliminate pore anomalies in the surface layer caused by the molding process or environmental exposure, the uppermost and lowermost slice layers were excluded from the analysis (the average value of the core region is indicated by a dotted line in the figure). This methodological approach ensures that the results obtained more accurately reflect the true pore characteristics of the specimen’s core region. The results demonstrate that the porosity of each cross-section exhibits significant spatial non-uniformity and random fluctuation, which is closely related to the distribution of aggregate, the degree of hydration reaction, and the uniformity of vibration. The porosity of the core area of specimens A1, A2, and A3 is found to be distributed at a consistent rate of 20%, 26%, and 31%, respectively. Despite local fluctuations, the overall distribution trend is highly consistent with the results of the slicing method, thereby confirming the hypothesis that both CT and slicing methods can effectively reflect the characteristics of the internal pore structure of the specimens.

## 4. Discussion

Pore characteristics are among the primary factors influencing the macroscopic properties of vegetated concrete, and there exists a specific functional relationship between the pertinent parameters and performance indicators such as compressive strength and water permeability coefficient. A substantial body of research has been dedicated to the examination of the correlation between porosity and macroscopic performance. However, it is imperative to acknowledge that other pore structure characteristics, including but not limited to equivalent pore size and fractal dimension, have been shown to exert a substantial influence on performance. Therefore, function fitting was performed in order to analyse the relationship between these different pore characteristics and both compressive strength and the water permeability coefficient.

### 4.1. Porosity and Compressive Strength, Permeability Coefficient

For ordinary concrete, compressive strength is the core design parameter, and porosity is typically kept low to ensure mechanical properties. For vegetated concrete, however, the primary functional objective is to provide a connected pore network that is conducive to plant root growth and water infiltration. Compressive strength is only employed as a constraint to ensure structural stability during the project’s operational phase, rather than as a core design objective. In this context, low compressive strength is an expected characteristic of vegetated concrete. The focus of material design is therefore not on achieving ultra-high strength, but on finding the optimal balance: maximising the material’s ecological function while meeting the minimum strength requirements for engineering applications.

For the engineering application of water conservancy ecological slope protection, this study proposes a clear threshold for vegetated concrete: continuous porosity must be ≥20% to allow for root penetration and growth, and the 28-day compressive strength must be ≥5 MPa to withstand rain erosion and prevent slope destabilisation. Test results demonstrate that the negative correlation between porosity and compressive strength in vegetated concrete remains significant: a 10% increase in porosity results in a 20% decrease in compressive strength, consistent with the fundamental principles of porous concrete materials. Furthermore, the correlation between connected porosity and compressive strength is stronger than that of cross-sectional porosity. This finding is significant for the practical design of vegetated concrete: cross-sectional porosity contains a large number of closed pores that cannot provide space for root growth or water infiltration. Focusing solely on controlling total porosity leads to an imbalance between ecological function and mechanical properties. Using the specimens in this study as an example, controlling the connected porosity at 20–30% ensures that the compressive strength of all the specimens meets the minimum requirement of 5 MPa. Of these specimens, those with an aggregate size of 10–15 mm can achieve a higher level of strength while still meeting the porosity requirement. This makes them more suitable for slope protection projects that require high structural stability. For projects requiring better vegetation growth and water permeability, specimens with an aggregate size of 20–25 mm can provide higher connected porosity and a more reasonable pore size distribution. When the target porosity does not exceed 25%, the compressive strength can still exceed 5 MPa, meeting the basic project stability requirements.

**Figure 10:**
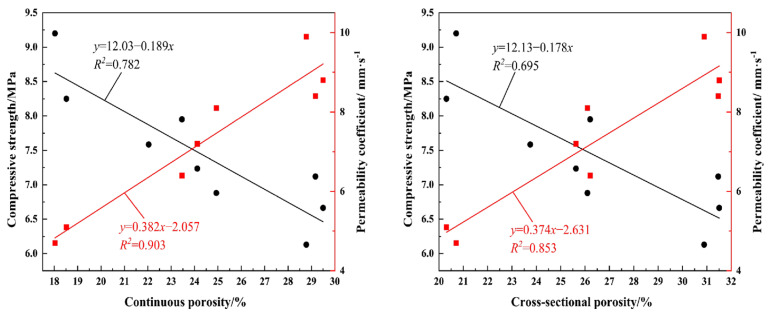
Fitting Curves for Porosity vs. Compressive Strength and Permeability Coefficient.

Conversely, there is a significant positive correlation between effective connectivity porosity and permeability coefficient, the latter being the core embodiment of the ecological function of vegetated concrete. Unlike cross-sectional porosity, which has a weak correlation with the permeability coefficient, connected porosity can more accurately predict the macroscopic permeability behaviour of the material because it directly reflects the topological characteristics of the three-dimensional connected pore network and the complexity of the seepage channels within the material. This further confirms that connected porosity is a central parameter for achieving a balance between strength and permeability in vegetated concrete: it is a decisive indicator of the material’s core ecological function, as well as a key parameter that influences its mechanical properties. As shown in Figure 10.

### 4.2. Equivalent Pore Size and Compressive Strength, Permeability Factor

**Figure 11:**
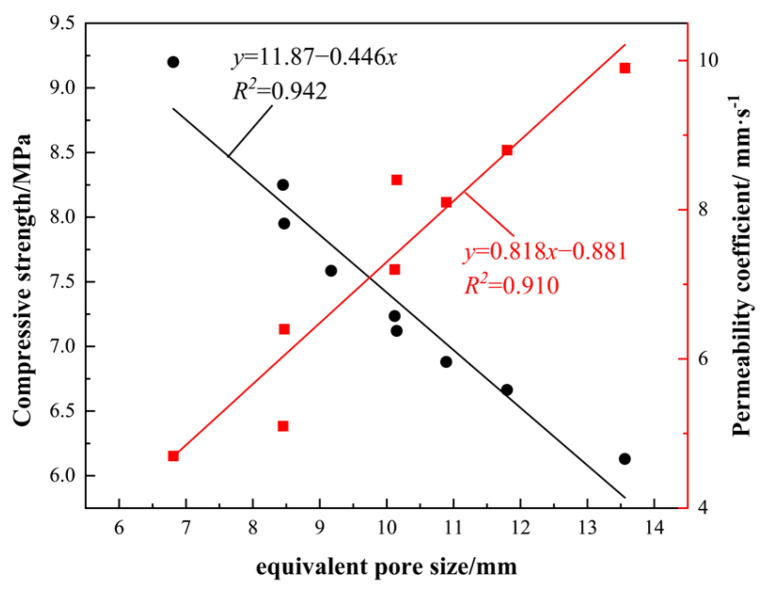
Equivalent Pore Diameter vs. Compressive Strength and Permeability Coefficient Fitting Curves.

A strong correlation exists between equivalent pore size and both compressive strength and the water permeability coefficient, as demonstrated in Figure 11. An increase in the equivalent pore diameter of the vegetated concrete results in an augmentation of the size of the internal permeable channels and an escalation in the proportion of through-channels in the pore network. This process has been shown to result in the expansion of the effective hydraulic radius, a reduction in the resistance to water penetration within the concrete, and a significant improvement in its water permeability. When the equivalent pore size ranged from 6 to 14 mm, the compressive strength of the vegetated concrete decreased as the equivalent pore size increased. The increase in equivalent pore size has been shown to result in a significant decrease in the encapsulation density of the paste around the aggregate. This, in turn, has been demonstrated to weaken the connection between aggregates and to reduce the effective transmission of force. Moreover, the augmentation in the dimensions and quantity of internal permeable channels and analogous pores engenders defects in the transmission of internal forces, culminating in diminished strength in comparison to vegetated concrete characterised by more diminutive pore sizes.

The size of the equivalent pore diameter exerts a significant influence on the performance of vegetated concrete. It has been demonstrated that larger equivalent pore sizes have a detrimental effect on compressive strength, whilst concomitantly resulting in a substantial increase in water permeability. This performance trade-off must be carefully weighed in practical applications; the appropriate equivalent pore size must be selected according to specific project requirements to achieve the best results.

### 4.3. Fractal Dimension and Compressive Strength, Permeability Coefficient

**Figure 12:**
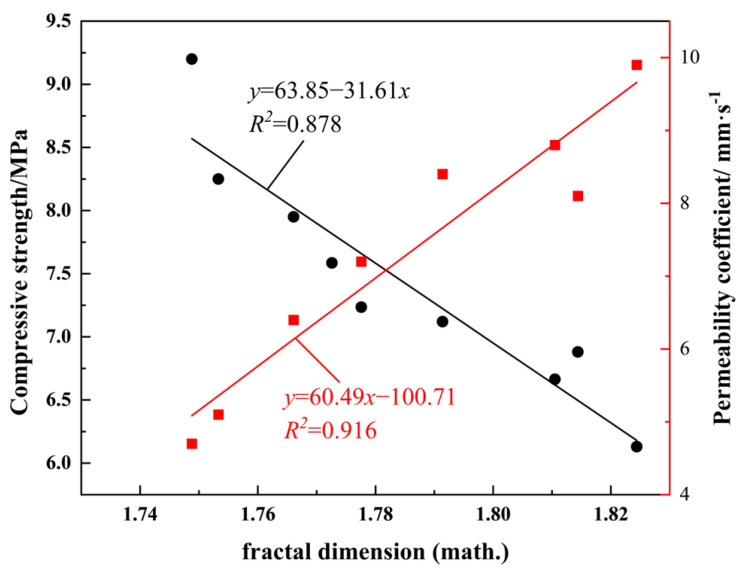
Fractal Dimension vs. Compressive Strength and Permeability Coefficient Fitting Curves.

The fractal dimension has been demonstrated to describe the relationship between pore structure and both compressive strength and the permeability coefficient, as illustrated in Figure 12. A negative correlation has been demonstrated between fractal dimension and compressive strength; as the fractal dimension increases, the compressive strength exhibits a decreasing trend. In accordance with the principles of fractal theory, an augmentation in fractal dimension gives rise to a more complex pore distribution, more irregular pore morphology, and increased pore space occupancy. This phenomenon leads to a reduction in the densification of the vegetated concrete structure, consequently weakening its compressive capacity.

In contrast, a positive correlation is observed between fractal dimension and the water permeability coefficient. Fractal dimension, as a key index for quantifying the self-similarity of the pore network and the efficiency of space filling, is important for the enhancement of water permeability. An increase in fractal dimension is indicative of enhanced self-similarity in the pore structure, which optimises the topology of the pore network, facilitating the penetration and flow of water and thereby enhancing the material’s water permeability. It is generally accepted that a higher fractal dimension corresponds to a more complex and spatially distributed pore structure. This, in turn, has been shown to promote the formation of penetrating pore channels, thereby effectively reducing flow resistance during fluid infiltration. This, in turn, enhances overall fluid permeability.

The correlation between the fractal dimension of the pore structure and both the water permeability coefficient and the compressive strength of vegetated concrete specimens has been demonstrated, thereby reinforcing the association between the macro- and microscopic properties of the specimens and fractal geometry. The present study proposes a novel methodology for conducting a more profound investigation into the characteristics of vegetated concrete specimens.

## 5. Conclusions

The present study investigated the pore structure characteristics of vegetated concrete and their correlation with macroscopic properties. This investigation revealed the intrinsic correlation and mechanism of action between aggregate particle size, pore structure characteristic parameters, and material properties. The primary conclusions that can be drawn from this analysis are as follows:

(1) The porosity of vegetated concrete has been shown to have a significantly negative correlation with its compressive strength. For every 10% increase in porosity, the compressive strength is known to decrease by approximately 20%. Increasing aggregate particle size has been shown to reduce strength, but to significantly improve water permeability. In the case of large-sized aggregates with a low target porosity, slurry sedimentation is probable, resulting in a measured porosity that exceeds the design value.
(2) Deepened physical interpretation of fractal dimension: Fractal dimension was demonstrated to serve as an integrated descriptor of pore complexity, surface roughness, and network connectivity. The negative correlation with compressive strength (R^2^ = 0.91) is physically interpreted as the effect of stress concentration at irregular pore boundaries, while the positive correlation with permeability (R^2^ = 0.88) reflects enhanced flow pathways in tortuous networks. Thus, fractal dimension effectively quantifies the inherent strength–permeability trade-off in vegetated concrete, providing a theoretical basis for using fractal analysis as a predictive tool in material design.
(3) Elucidation of aggregate size regulation mechanism: The study quantitatively revealed how aggregate particle size modulates pore structure evolution—including pore size distribution, connectivity, and fractal characteristics—and consequently influences macroscopic performance. Larger aggregates were shown to promote pore interconnection and increase fractal dimension, enhancing permeability but reducing strength. This provides practical guidance for optimizing mix proportions to achieve desired performance targets.
(4) Validation of 2D image analysis reliability: CT scans showed that the deviation of the 3D total porosity from that obtained by the 2D slicing method was less than 1%, verifying the reliability of the 2D method for routine porosity assessment.
(5) Development of quantitative regression models: Multi-parameter regression models were established linking pore structure parameters to macroscopic performance, providing a quantitative basis for performance prediction and mix design optimization.

## Figures and Tables

**Table 4:** Weight of Each Component of Reinforcing Agent for Pervious Concrete.

Nano-Sized Silicon Powder	Water Reducing Agent	Cellulose	Calcium Sulfate Whiskers	Trisodium Phosphate	Calcium Carboxylate	Alkali Metal Carbonate	Carboxamide
50~80	10~15	2~4	8~10	5~8	1.5~5	2~6	0.5~2

**Table 5:** Vegetated Concrete Mix Proportions.

Number	Coarse Aggregate Particle Size (mm)	Porosity (%)	Coarse Aggregate (kg·m^−3^)	Cement (kg·m^−3^)	Fly Ash (kg·m^−3^)	Water (kg·m^−3^)
A1	10–15	20%	1567	259	91	105
A2		25%	1567	203	71	82
A3		30%	1567	147	52	60
B1	15–20	20%	1529	280	99	114
B2		25%	1529	224	79	91
B3		30%	1529	169	59	68
C1	20–25	20%	1523	295	104	120
C2		25%	1523	240	84	97
C3		30%	1523	184	65	74

**Table 6:** Statistical Summary of Parameters for Vegetated Concrete.

Number	Design Porosity (%)	Continuous Porosity (%)	Cross-Sectional Porosity (%)	Equivalent Aperture (mm)	Number of Pores	Fractal Dimension
A1	20	19.03	20.71	6.81	88	1.7488
A2	25	24.46	26.21	8.47	59	1.7661
A3	30	29.87	31.47	10.15	54	1.7914
B1	20	19.40	20.31	8.45	60	1.7533
B2	25	24.42	25.63	10.12	48	1.7776
B3	30	29.70	31.51	11.80	43	1.8105
C1	20	22.04	23.75	9.17	58	1.7726
C2	25	24.93	26.10	10.89	38	1.8144
C3	30	28.78	30.89	13.56	31	1.8244

## Data Availability

The original contributions presented in this study are included in the article. Further inquiries can be directed to the corresponding author.
